# Association of heart failure and its comorbidities with loss of life expectancy

**DOI:** 10.1136/heartjnl-2020-317833

**Published:** 2020-11-05

**Authors:** Michael Drozd, Samuel D Relton, Andrew M N Walker, Thomas A Slater, John Gierula, Maria F Paton, Judith Lowry, Sam Straw, Aaron Koshy, Melanie McGinlay, Alexander D Simms, V Kate Gatenby, Robert J Sapsford, Klaus K Witte, Mark T Kearney, Richard M Cubbon

**Affiliations:** 1 Leeds Institute of Cardiovascular and Metabolic Medicine, University of Leeds, Leeds, UK; 2 Leeds Institute of Health Sciences, University of Leeds, Leeds, UK; 3 Department of Cardiology, Leeds General Infirmary, Leeds, UK

**Keywords:** heart failure with reduced ejection fraction, heart failure

## Abstract

**Objective:**

Estimating survival can aid care planning, but the use of absolute survival projections can be challenging for patients and clinicians to contextualise. We aimed to define how heart failure and its major comorbidities contribute to loss of actuarially predicted life expectancy.

**Methods:**

We conducted an observational cohort study of 1794 adults with stable chronic heart failure and reduced left ventricular ejection fraction, recruited from cardiology outpatient departments of four UK hospitals. Data from an 11-year maximum (5-year median) follow-up period (999 deaths) were used to define how heart failure and its major comorbidities impact on survival, relative to an age–sex matched control UK population, using a relative survival framework.

**Results:**

After 10 years, mortality in the reference control population was 29%. In people with heart failure, this increased by an additional 37% (95% CI 34% to 40%), equating to an additional 2.2 years of lost life or a 2.4-fold (2.2–2.5) excess loss of life. This excess was greater in men than women (2.4 years (2.2–2.7) vs 1.6 years (1.2–2.0); p<0.001). In patients without major comorbidity, men still experienced excess loss of life, while women experienced less and were non-significantly different from the reference population (1 year (0.6–1.5) vs 0.4 years (−0.3 to 1); p<0.001). Accrual of comorbidity was associated with substantial increases in excess lost life, particularly for diabetes, chronic kidney and lung disease.

**Conclusions:**

Comorbidity accounts for the majority of lost life expectancy in people with heart failure. Women, but not men, without comorbidity experience survival close to reference controls.

## Introduction

Chronic heart failure (CHF) is a common late phase in the natural history of many cardiovascular diseases, affecting millions of people globally, and remains associated with an appreciable mortality rate.^1^ In spite of declining age–sex adjusted incidence rates, the prevalence of heart failure continues to increase,^2^ reflecting improving survival rates and an ageing population. Hence, people with heart failure are increasingly old and have a rising burden of major comorbidity.^2^ These trends pose challenges for the estimation and communication of prognosis, with important implications for patients and clinicians aiming to make well-informed decisions. For example, established prognostication tools may be less reliable at predicting remaining life expectancy in people over 80 years^3^ and do not convey the substantial risk of death in similarly aged individuals without heart failure. Moreover, prognostic estimates do not describe the relative contribution of heart failure versus associated comorbidities, which may be important in defining therapeutic priorities in the growing population with multimorbidity. Indeed, non-cardiovascular causes of death are increasingly common in people with heart failure, especially with advancing age.^4 5^ Furthermore, prior research has shown substantial discordance between patient-predicted and prognostic model-predicted survival, illustrating the need to better communicate this important and sensitive topic.^6^ These issues suggest that alternate approaches to considering and communicating prognosis may be helpful for health professionals and people with heart failure. Therefore, we set out to describe the survival of people with heart failure *relative* to an age–sex matched control population and then define how comorbid disease contributes to the observed loss of survival.

## Methods

As described in our earlier publications,^4^ we conducted a prospective cohort study with the predefined aim of identifying prognostic markers in patients with CHF and reduced left ventricular ejection fraction (LVEF), receiving contemporary evidence-based therapy. This manuscript presents a post hoc analysis of the original study. Inclusion in the study required the presence of stable signs and symptoms of CHF for at least 3 months, age ≥18 years and LVEF ≤45% on transthoracic echocardiography. Between June 2006 and December 2014, consecutive patients attending specialist cardiology clinics (secondary and tertiary referral) in four UK hospitals were approached, and 1794 patients provided written informed consent. The Leeds West Research Ethics Committee gave ethical approval and the investigation conforms to the principles outlined in the *Declaration of Helsinki*.

Details of comorbid illness and symptomatic status (New York Heart Association (NYHA) classification) were collected by the recruiting physicians using medical history and care records at study enrolment, as we have previously described.^4^ Briefly, diabetes was defined on the basis of previous diagnosis and/or treatment with hypoglycaemic agents; chronic obstructive pulmonary disease (COPD) was defined on the basis of previous diagnosis and ischaemic aetiology was defined on the basis of detailed medical history and cardiac investigations (ECG, non-invasive imaging and coronary angiography, as appropriate). Venous blood was collected at study recruitment for assessment of renal function in the local hospital chemical pathology laboratories. Estimated glomerular filtration rate (eGFR) was calculated using the Modification of Diet in Renal Disease method, with chronic kidney disease (CKD) stage 4 or worse being defined as eGFR <30 mL/minute/1.73 m^2^.^7^ Two-dimensional echocardiography was performed according to the American Society of Echocardiography recommendations.^8^ Resting heart rate was measured using 12-lead ECGs. Prescribed doses of loop diuretics, ACE inhibitors (ACEi), angiotensin receptor blockers (ARB) and β-adrenoceptor antagonists (β-blockers) were collected at study recruitment. Total daily doses of β-blockers, ACEi (or ARB if used instead of ACEi) and loop diuretic were expressed relative to the maximal licensed dose of bisoprolol, ramipril and furosemide, respectively, as previously published.^4^ Receipt of cardiac resynchronisation therapy or implantable cardioverter defibrillator was assessed during the 6-month period after recruitment.

All patients were registered with the UK Office of Population Censuses and Surveys, which provided details of time of death, with a final censorship date of 8 November 2018; maximum follow-up was for 11 years. Actuarial survival predictions were derived from the UK National Life Tables (UK-NLT), an official survival estimation measure produced by the UK government.^9^ The UK-NLT provide annual death rates by sex and age for overlapping 3-year periods, which we assigned the value to the middle of the range: for example, the death rate for 2011–2013 is used with patients recruited in 2012. This provides the baseline survival for members of the public with this age and sex, which we used as a reference control population.

### Statistics

Patient characteristics are reported using the mean and SD for continuous variables, with categorical variables summarised using the count of each class and the percentage of the dataset it represents. Median survival rates and Kaplan-Meier curves describing the observed cohort survival, stratified by sex, were produced using the survival package in R (https://CRAN.R-project.org/package=survival). Relative survival data were produced using the relsurv package within R (https://www.jstatsoft.org/article/view/v087i08) and illustrated with both expected mortality (the age–sex matched mortality rate in the general population defined by the UK-NLT) and excess mortality (the mortality rate in our study cohort after removing age–sex matched mortality rate in the general population) curves. In particular, we investigated the excess loss of life associated with heart failure, both in the entire cohort, stratified by sex, and according to the number of comorbidities. Wald CIs are used for mortality rate, while 500 bootstrap samples are used to produce CIs for years of life lost, with t-tests to compare the means between sexes.

To investigate the independent impact of comorbidities, a multiplicative relative survival model was produced using the relsurv package within R. The presence of four major comorbidities (COPD, diabetes, ischaemic aetiology, CKD grade ≥4) were used as independent variables, in addition to the LVEF. These four comorbidities were chosen as they are included in large validated heart failure prognostication tools (the Seattle Heart Failure Model (SHFM) and the Meta-analysis Global Group in Chronic Heart Failure score (MAGGIC),^10 11^ implying likely association with loss of life expectancy. Excess HRs (EHRs) and Wald CIs are reported and fitted using the maximum likelihood principle. The excess hazard due to heart failure is modelled as a constant as our sensitivity analyses revealed this did not vary over time.

## Results

**Figure 1:**
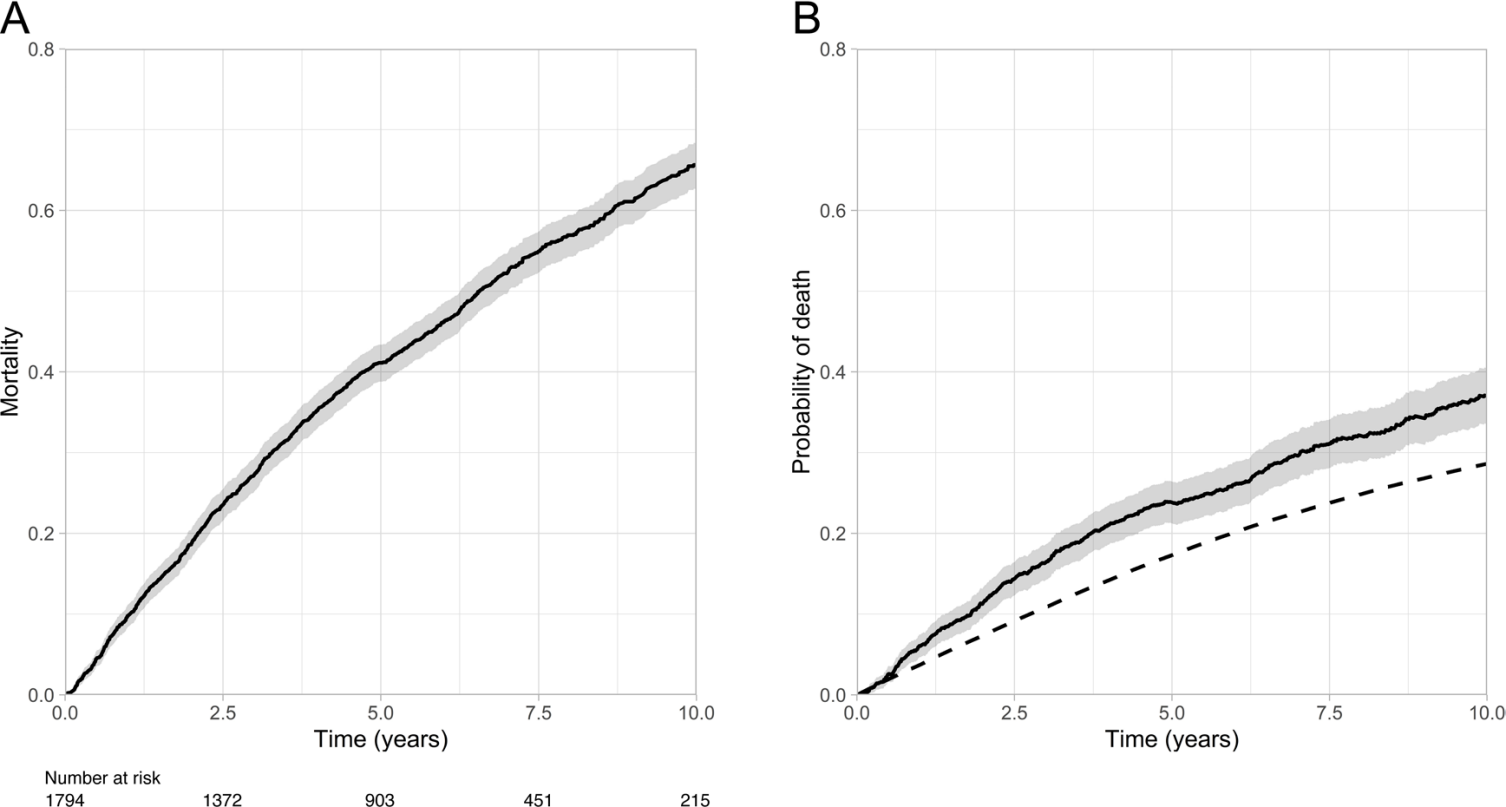
Absolute and relative survival of the study cohort. (A) Kaplan-Meier curve illustrating observed mortality in the study cohort; (B) expected mortality rate (dashed line) and excess mortality rate (continuous line with shaded 95% CI) in an age-sex matched UK population.

As described in table 1, the study cohort had a mean age of 69.6 years and 73% were male. The aetiology of heart failure was ischaemic heart disease in 59% of cases, mean LVEF was 32% and 31% of people had moderate to severe dyspnoea (NYHA classification 3 or 4). Major comorbidity was common, with diabetes being present in 28%, COPD in 16% and CKD grade ≥4 in 18%. After a maximum follow-up period of 11 years (median 5 years), 999 (55.7%) deaths occurred. As illustrated in figure 1A, median survival was 6.6 years (95% CI 6.3 to 7 years). However, this illustrates a composite of the excess risk of death in this cohort *plus* the background risk in the general population, which is likely to be substantial in the context of their advanced age. To address this, we constructed relative survival models that define the expected mortality in the background population (figure 1B), and contrast this with the observed mortality in our study cohort to define their excess mortality risk (figure 1B). After 10 years, the expected background population mortality rate is 28.6% (95% CI 27.8%–29.4%); in addition to this, our study cohort experienced an excess risk of 37% (95% CI 33.6% to 40.5%). Expressed as years of life lost over 10 years of follow-up, the expected loss accounts for 1.6 (95% CI 1.54 to 1.72) years, while the excess risk accounts for a further 2.2 (95% CI 1.99 to 2.41) years, resulting in a cumulative loss of 3.8 (95% CI 3.66 to 4.0) years. Therefore, our study cohort lost 2.4-fold (95% CI 2.2 to 2.5) more life than expected.

**Table 1:** Participant characteristics

	Total cohort (N=1794)	Men (n=1311)	Women (n=483)	P value
Age (years)	69.6 (12.5)	69.3 (12.1)	70.4 (13.5)	0.1
Ischaemic aetiology (n (%))	1064 (59.3)	835 (63.7)	229 (47.4)	<0.001
Diabetes (n (%))	504 (28.1)	384 (29.3)	120 (24.8)	0.06
COPD (n (%))	283 (15.8)	195 (14.9)	88 (18.2)	0.09
CKD 4 or above (n (%))	141 (7.9)	86 (6.6)	55 (11.4)	0.001
NYHA class 3/4 (n (%))	551 (30.7)	386 (29.5)	165 (34.2)	0.06
LV ejection fraction (%)	32 (9.5)	31.7 (9.5)	32.6 (9.5)	0.08
Beta blocker use (n (%))	1516 (84.7)	1117 (85.5)	399 (82.6)	0.14
QRS interval (ms)	123.2 (31)	125 (30.9)	118.1 (30.7)	<0.001
Bisoprolol equivalent dose (mg/day)	3.9 (3.4)	4 (3.4)	3.5 (3.3)	0.01
ACEi or ARB use (n (%))	1618 (90.4)	1195 (91.4)	423 (87.6)	0.014
Ramipril equivalent dose (mg/day)	4.9 (3.5)	5.1 (3.6)	4.3 (3.4)	<0.001
MRA use (n (%))	684 (38.2)	507 (38.8)	177 (38.8)	0.41
Furosemide equivalent dose (mg/day)	51 (50)	52 (52)	49 (43)	0.18
CRT (n (%))	452 (25.2)	353 (26.9)	99 (20.5)	0.005
ICD (n (%))	209 (11.6)	184 (14)	25 (5.2)	<0.001

Continuous data displayed as mean (SD) and categorical data as n (%).

ACEi, ACE inhibitor; ARB, angiotensin receptor blocker; CKD, chronic kidney disease; COPD, chronic obstructive pulmonary disease; CRT, cardiac resynchronisation therapy; ICD, implantable cardioverter defibrillator; LV, left ventricular; MRA, mineralocorticoid receptor antagonist; NYHA, New York Heart Association.

**Figure 2:**
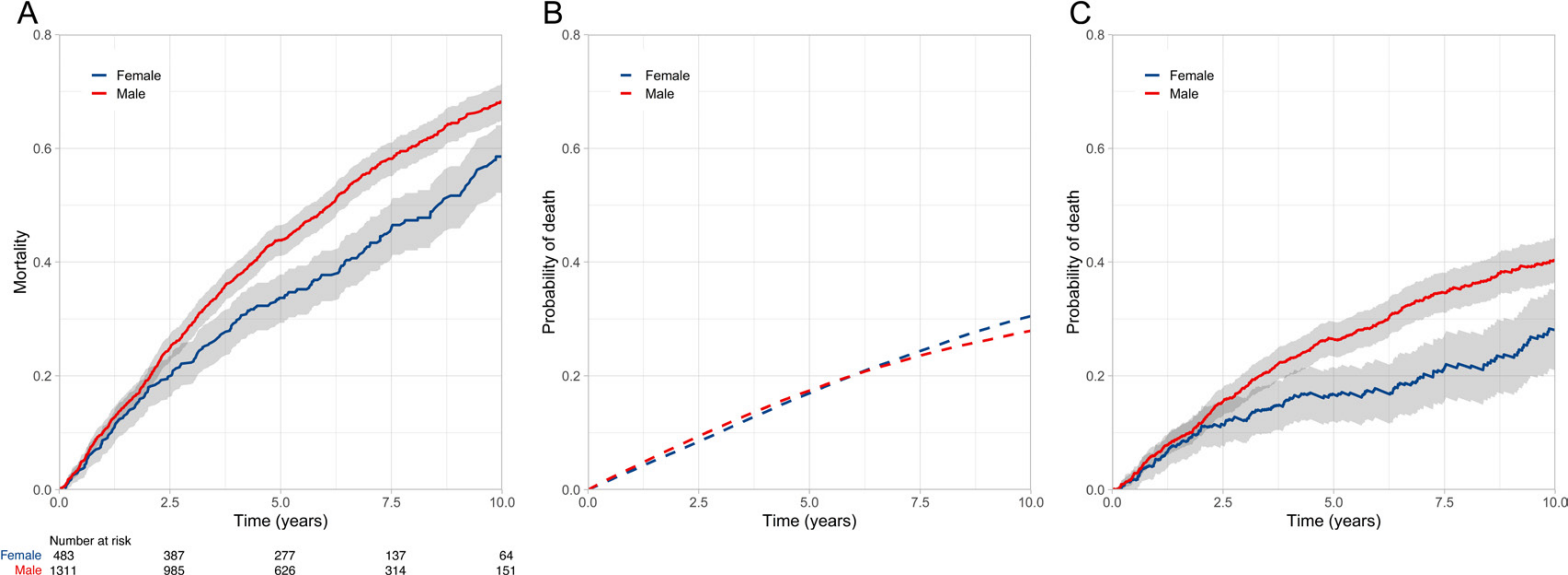
Absolute and relative survival stratified by gender. (A) Kaplan-Meier curve illustrating observed mortality of men and women in the study cohort; (B) expected mortality rate in an age-matched UK populations of men and women; (C) excess mortality rate in men and women from the study cohort relative to the age–sex matched UK populations, illustrating greater excess mortality in men than women with heart failure.

Next we explored the impact of male sex, given its established role as an adverse prognostic factor. As described for figure 1, the observed, expected and excess mortality of the cohort, stratified by sex, are shown in figure 2A–C. Men and women exhibited similar 10-year background mortality rates (27.9% (26.9%–28.9%) vs 30.5% (29%–32.1%)). However, excess 10-year mortality rates were higher in men than in women (40.3% (36.3%–44.2%) vs 28% (21%–35.1%)). Over 10-years of follow-up, the background loss of life was 1.6 years in both men and women, but the excess risk was 2.4 (95% CI 2.2 to 2.7) years in men versus 1.6 (95% CI 1.2 to 2.0) years in women, resulting in an average cumulative loss of 4 and 3.2 years, respectively. Therefore, men and women lost 2.5-fold (95% CI 2.3 to 2.7) and twofold (95% CI 1.7 to 2.3) more life than expected, respectively, suggesting male sex is associated with a higher risk of heart failure phenotype (p<0.001).

**Figure 3:**
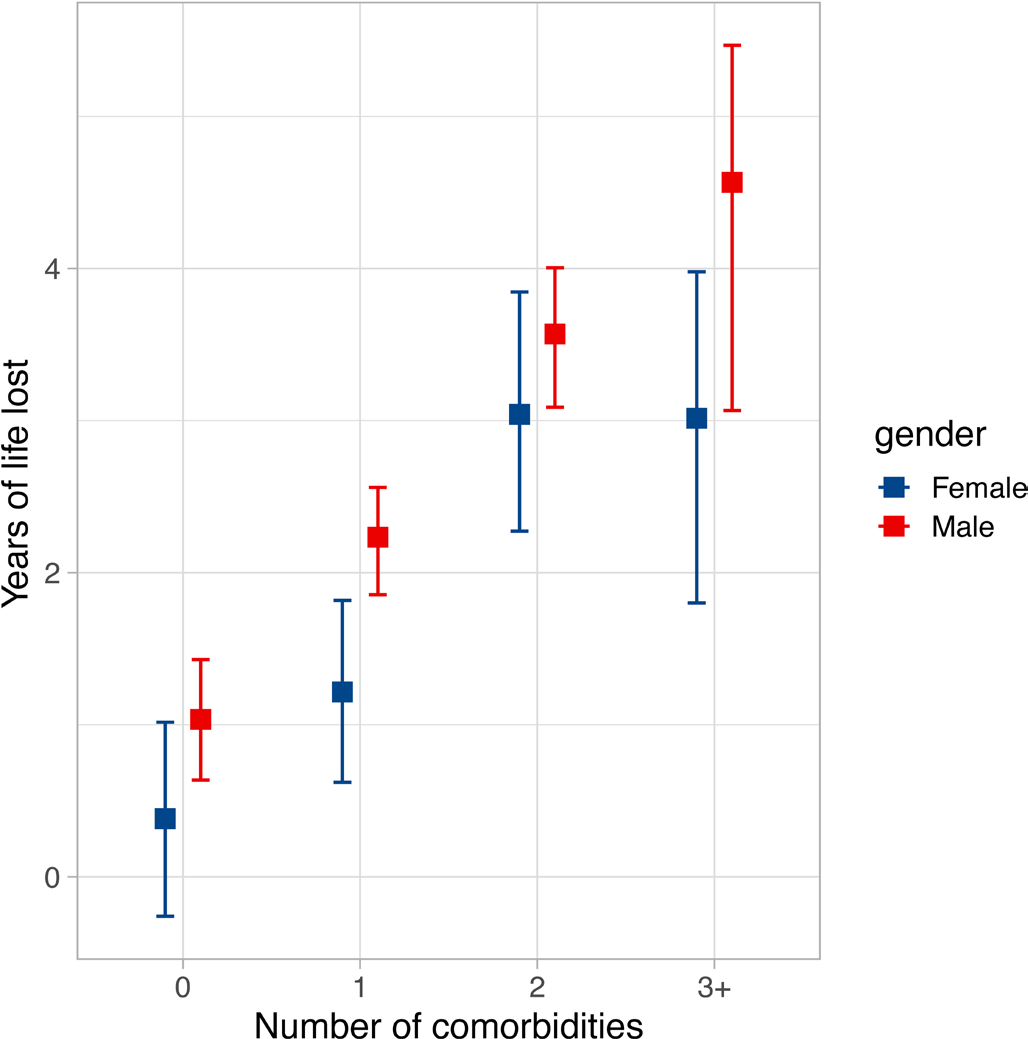
Loss of expected life according to sex and number of comorbidities. Loss of expected life over 10 years of follow-up, with 95% CI, in men (red) and women (blue) according to number of comorbidities (from ischaemic heart disease, chronic obstructive lung disease, diabetes and chronic kidney disease stage 4 or above).

Given the differing comorbidity profile of men and women (table 1), we next explored how they might contribute to the differential loss of expected life in these groups. As illustrated in figure 3, men and women with increasing numbers of comorbidities experienced substantially greater loss of life expectancy. Indeed, in patients with three or more comorbidities, men lost an excess of 4.6 years (95% CI 3.1 to 5.5), while women lost an excess of 3.1 years (95% CI 1.9 to 4). Importantly, in patients without major comorbidity, men still experienced excess loss of life, while women experienced less and were non-significantly different from the reference population (1 year (95% CI 0.6 to 1.5) vs 0.4 years (95% CI −0.3 to 1); p<0.001). To explore the contribution of specific comorbidities to loss of expected life, a multiplicative relative survival analysis was performed and the EHRs are presented in table 2. All these were associated with loss of expected life, but with substantial heterogeneity in their effect size. Higher LVEF was also associated with modest reductions in loss of expected life. Notably, while statistically significant, the baseline EHR was just above 1 and approximately constant for the duration of the study; this implies that the excess risk associated with heart failure per se remained broadly constant. Moreover, sensitivity analyses using various approaches to allow time variance in the baseline excess hazard did not reveal differences in the EHRs of the main comorbidities.

**Table 2:** Multivariate survival analysis

	EHR	95% CI of EHR		P value
		Low	High	
Diabetes	1.61	1.40	1.84	<0.001
COPD	1.79	1.53	2.09	<0.001
Ischaemic aetiology	1.06	0.93	1.22	0.386
CKD 4 or above	1.62	1.33	1.99	<0.001
LVEF (per % increase)	0.98	0.97	0.98	<0.001
Baseline	1.00	1.00	1.00	<0.001

Excess HRs describe risk of reduced life expectancy relative to actuarial projections.

CKD, chronic kidney disease; COPD, chronic obstructive pulmonary disease; EHR, excess HR; LVEF, left ventricular ejection fraction.

## Discussion

By considering survival relative to actuarial estimates of life expectancy, we have shown that heart failure is associated with a 2.4-fold greater loss of time alive than observed in the age–sex matched general population over 10 years. Notably, male sex and accrual of major comorbidities are associated with larger loss of life, while women without major comorbidity have life expectancy compatible with actuarial projections. Lower LVEF was also associated with greater excess loss of life. This approach to defining survival may provide useful perspective for clinicians considering the magnitude of risk posed by heart failure in the context of an ageing and increasingly multimorbid population. This context may be particularly useful when communicating risk to people with heart failure, who often struggle to estimate their own prognosis.

### Estimating prognosis

Validated tools, such as SHFM and MAGGIC,^10 11^ are already available to estimate the prognosis of people with heart failure in terms of absolute lifespan. While valuable, it is important to ask whether this approach tells patients and clinicians what they want to know. By overlooking the inevitability of death in similar people without disease, such prognostic estimates may be misinterpreted, resulting in poorly informed decision-making. The challenges of prognostication in people with heart failure are illustrated by the discordance between model-estimated and patient-estimated absolute life expectancy.^6^ By considering survival relative to actuarially predicted life expectancy, we hope that our approach will provide essential context to aid the challenging process of communicating prognosis. This may take the form of ‘ballpark’ estimates of excess loss of life for groups of similar people or by developing an individualised prognostication tool, such as the SHFM. Further research is needed to address the validity, acceptability and added value of this approach, but we think that it has the potential to improve prognostication in clinical practice.

### Multimorbidity as risk marker and therapeutic target

Recent research describing all people with heart failure in a representative cohort of 4 million UK residents found that multimorbidity is becoming increasingly common.^2^ While we focused on just four major comorbidities, 26% of our cohort were not multimorbid (ie, heart failure with at least one comorbidity), and 31% had two or more of these comorbidities. Strikingly, people with three or more comorbidities experienced approximately fivefold greater excess loss of life than people with no comorbidity (figure 3). While we selected comorbidities known to contribute to prognostication in people with heart failure, this confirms that the accumulation of comorbidity is an important part of lost life expectancy in heart failure; it will be important for further research to study the additional impact of other common comorbidities. Optimal medical therapy is associated with substantial reductions in heart failure morbidity and lifespan extension in clinical trial participants,^12^ yet clinical trials often exclude multimorbid people. These data highlight the need to design clinical trials specifically recruiting people with heart failure and multimorbidity, possibly applying complex interventions that target more than just the heart failure syndrome.

### Heart failure in men and women

Poorer survival of men has been observed in many studies of heart failure and is accounted for in the SHFM and MAGGIC prognostic models.^10 11^ While this could to some extent be attributed to differences in comorbidity, such as ischaemic heart disease, our observations from people with heart failure and no major comorbidity still show clear differences in the outcomes of men and women. Notably, the survival of women without major comorbidity overlapped with that of the matched general population (figure 3). The mechanisms of this sexual dimorphism remain debated,^13 14^ but it is clear that clinical trials and guidelines should carefully consider the differences between men and women with heart failure.

### Limitations

Although our work has key strengths, it is important to acknowledge limitations that should be addressed by ongoing research. First, we have deliberately chosen not to derive an individualised risk assessment tool; however, our methods could easily be used to extend the data provided by individualised prognostic models, such as SHFM and MAGGIC.^10 11^ It will also be important to understand whether healthcare professionals and patients find survival estimates relative to actuarial life expectancy more useful than absolute survival estimates. Next, our data should not be generalised to other populations (eg, heart failure with preserved ejection fraction or patients not attending specialist heart failure clinics), but our methods could easily be applied to published datasets. It is also important that our 11-year follow-up period represents a modest proportion of predicted life expectancy in our youngest participants, and our cohort is relatively old, so caution should be applied in extrapolating our data to the youngest people with heart failure. Moreover, we were unable to account for the likely accrual of comorbidity over time, which may have led to underestimation of their association with excess mortality. Finally, it is important to note that our expected survival data are derived from the UK general population which will include some people with heart failure; therefore, loss of expected survival is in relation to the age–sex matched general population, not an age–sex matched heart failure free population.

## Conclusions

By framing survival in the context of actuarial predictions, we have shown that people with heart failure with reduced LVEF lose 2.4-fold more of life than expected. However, most of this loss of life expectancy is accounted for people with comorbidity, particularly in women. Our work provides a different framework for clinicians and people with heart failure to consider prognosis and should prompt more focus on the issue of heart failure associated with complex multimorbidity.

**Key messages**

**What is already known on this subject?**

- Heart failure is associated with high mortality rates and estimates of prognosis often inform treatment decisions. However, survival estimates rarely consider what would be expected in a person of a similar age and sex without heart failure and so may be largely influenced by these factors, rather than heart failure and its comorbidities.

**What might this study add?**

- We defined the survival of people with heart failure relative to the age–sex matched UK population and show a marked increase in lost life expectancy as comorbidity accrues. Women, but not men, without comorbidity experienced survival close to the age–sex matched reference population.

**How might this impact on clinical practice?**

- Estimates of prognosis in people with heart failure should also consider what would be expected in the age–sex matched general population to focus on the disease-specific risks. Future clinical trials focused on improving survival in people with heart failure should focus on multimorbidity, possibly applying complex interventions that target more than just the heart failure syndrome.

## Data Availability

Data are available on reasonable request. The datasets generated and/or analysed during the current study are not publicly available due to inclusion of potentially identifying postal codes, but are available from the corresponding author on reasonable request.
